# A Solar-Enabled Strategy for Degrading Allura Red AC Using a Laddered Heterojunction Nanocomposite

**DOI:** 10.1021/acsomega.5c07920

**Published:** 2025-12-12

**Authors:** Manavi Yuvraj Suvarna, Antony Dasint Lopis, Khoobaram S. Choudhari, Pruthvi Y. D, Suresh D. Kulkarni

**Affiliations:** Manipal Institute of Applied Physics, 76793 Manipal Academy of Higher Education Manipal, Karnataka 576104, India

## Abstract

Allura Red AC is a synthetic azo dye that is widely used in the food and textile industries. It raises significant environmental and health concerns due to its chemical stability, toxicity, and resistance to conventional water treatment methods. In this study, we demonstrate the effective photocatalytic degradation of Allura Red AC using a solar-active nanocomposite consisting of Ag-coated Co^2+^-substituted ZnFe_2_O_4_ integrated with ZnO (Ag-CZFO/ZnO), synthesized via a microwave-assisted solvothermal method. The nanocomposite exhibited an extended light absorption up to 925 nm, enabling efficient utilization of both visible and near-infrared light. Under optimized conditions (pH 5.89, catalyst loading 1 g/L, dye concentration 50 μM), the system achieved 96% degradation of the dye within 150 min, with a pseudo-first-order rate constant of 0.0188 min^–1^. The introduction of electron and hole scavengers demonstrated that photogenerated holes played a crucial role in the oxidation process. Hydroxyl and superoxide radicals were also significant contributors to the degradation pathway, as confirmed by reactive oxygen species probe experiments. This process resulted in a substantial reduction of chemical oxygen demand from 31 to below 5 ppm, indicating nearly complete mineralization. Additionally, the photocatalyst maintained high activity over five reuse cycles, confirming its stability and potential for repeated use. Our laddered heterojunction solar photocatalyst (i) significantly reduced the charge recombination as observed by a 100-fold enhancement in the photocurrent and (ii) enhanced the charge transfer process as observed by the smallest semicircle in the Nyquist plots. These findings demonstrate that this solar-driven photocatalytic approach is a sustainable and effective method for degrading persistent organic pollutants such as Allura Red AC. This offers a promising solution for the large-scale treatment of dye-contaminated wastewater using sunlight as an energy source.

## Graphical abstract

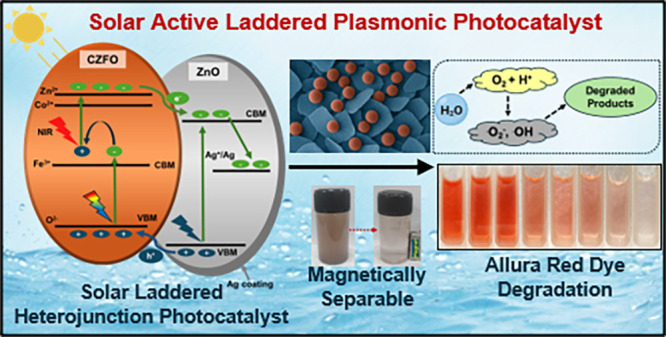

## 1 Introduction

The pursuit of a better standard of living has driven rapid industrialization, resulting in the generation of vast amounts of wastewater.^1^ Improving wastewater management is essential for tackling water and environmental challenges, reducing clean water costs, and preventing the spread of waterborne diseases for healthier communities.^2^ Synthetic food color dyes are often preferred over natural colorants due to their widespread availability, lower production costs, and longer shelf life. Their use in enhancing visual appeal is common in the food processing and textile industries.^3^ Poor management of wastewater is increasingly concerning, as an estimated 5–10% of dyes used in industries such as textiles, paper, and cosmetics are released as industrial waste.^4^ Annually, about 280,000 tons of textile dyes are discharged into the environment through industrial wastewater.^5,6^ Dumping excessive dye-containing wastewater into water bodies has resulted in significant contamination. These dyes can severely disrupt aquatic ecosystems, ultimately disturbing the food chain and threatening biodiversity.^7,8^

Allura Red AC (ALR) (C_18_H_14_N_2_Na_2_O_8_S_2_, E129), a water-soluble anionic azo dye, is widely used for its intense red hue and physicochemical stability. Its IUPAC designation is Disodium 6-hydroxy-5-[(2-methoxy-5-methyl-4-sulfonatophenyl)­diazenyl]­naphthalene-2-sulfonate. ALR is prevalent in bakery goods, dairy products (e.g., curd), pharmaceuticals, ice creams, and extensively in textile dyeing, particularly of acrylic yarn.^6,9^ According to regulatory guidelines, the acceptable concentration of ALR varies, depending on the food matrix. For soft drinks and other nonalcoholic beverages, the permissible range is typically 10–100 mg/L, whereas for sauces the allowable level is substantially higher, up to 500 mg/L. These limits are established to ensure consumer safety while maintaining the desired coloring effect in different food products.^10^ Owing to its broad usage, effluents containing ALR, especially when mixed with other industrial discharges, are a significant contributor to the heightened toxicity of industrial wastewater.^11–13^ ALR contamination is frequently detected in surface waters, correlated with proximity to wastewater discharge sites. The dye’s persistence enables it to leach into groundwater and accumulate in soils, thereby degrading soil quality and entering the food web, which raises pressing ecological and public health concerns.^14,15^

Although ALR exhibits low acute toxicity at minimal exposure, its pervasive presence and resistance to environmental degradation pose substantial long-term risks. Bioaccumulation from chronic exposure has been associated with hyperactivity disorders and allergic reactions, notably in children.^16,17^ The recalcitrant nature of ALR and related azo dyes has prompted the development of numerous treatment strategies, including adsorption,^18^ membrane separation,^19^ ozonation,^20^ electrochemical methods,^21^ and advanced oxidation processes (AOPs).^22^ Among these, AOPs characterized by the in situ generation of highly reactive species such as hydroxyl radicals are particularly effective for mineralizing dyes to less harmful products.^23,24^

Photocatalysis, a subset of AOPs, represents a sustainable, cost-effective approach for wastewater remediation by employing semiconductor nanomaterials as photocatalysts, which harness light (UV or visible) to generate reactive species that degrade organic pollutants.^25–27^ Conventional wide-band gap semiconductors such as TiO_2_ and ZnO are limited to UV activation, utilizing less than 5% of the solar spectrum, which restricts their solar-driven efficiency and increases operational costs. Consequently, considerable effort has focused on engineering visible-light-active photocatalysts to exploit a broader fraction of solar energy. Plasmonic copper nanoparticles with WO_3–*X*_@ZNC were used for visible and IR photodegradation of ciprofloxacin. Photocatalytic activity was due to plasmonic hot electrons and hydroxyl radicals.^28^ Au-decorated ZnO nanosheet arrays were employed for Methyl Orange photodegradation under visible light. Au presence showed improved degradation owing to a nonlinear enhancement of hot electron injection and plasmonic degradation.^29^ A visible-light-active plasmonic photocatalyst Au/WO_3_ was utilized for the photodegradation of Rhodamine B and Methylene Blue. The enhanced optical/electrical properties were attributed to the plasmonic effect of gold.^30^ Au NPs on g-C_3_N_4_ nanophotocatalyst with visible light activity were used for Rhodamine B degradation. They reported an enhancement in the charge separation, better light absorption, and a decrease in the recombination rate of the electron–hole pairs due to the presence of gold on g-C_3_N_4_.^31^ Zinc ferrite (ZnFe_2_O_4_) is a promising candidate in this domain due to its nontoxic nature, chemical robustness, and intrinsic magnetism, enabling facile recovery and reusability.^32^ Nevertheless, narrow-band gap semiconductors, though responsive to visible light, often suffer from rapid recombination of photogenerated electron–hole pairs, thereby limiting photocatalytic efficacy. To counteract this, heterojunction architectures, such as ZnFe_2_O_4_/ZnO, have been devised to facilitate efficient charge separation, resulting in enhanced photocatalytic performance and extended light absorption into the near-infrared (NIR) region.^33^ Formation of heterojunction has evolved as a useful strategy to (i) regulate the photogenerated charge carrier separation and migration processes, (ii) reduce the activation energy required for photocatalytic reaction, (iii) enhance the carrier lifetime, and (iv) form an electric double layer structure with an edge over a single photocatalyst for better separation of photogenerated charge carriers.^34,35^ Photodegradation using heterojunction photocatalysts has been explored widely, for example, (i) degradation of a mixture of methylene blue and methyl orange using BiVO_4_/CeO_2_, a type-II heterojunction photocatalyst^36^ and (ii) degradation of ALR dye using CaWO_4_/g-C_3_N_4_composite.^37^ We have reported visible light photodegradation of azo dye pollutants for the first time, using phase pure ZnFe_2_O_4_/ZnO core–shell nanoparticles synthesized using a combination of microwave and hydrothermal synthesis methods with good repeatability.^38^ However, without the addition of external electron scavengers (e.g., H_2_O_2_), these heterojunctions typically display poor activity in the NIR, indicating that simple band gap modulation is insufficient for efficient sunlight utilization and may introduce extra costs and secondary pollution.

These limitations were addressed using Fe^2+^ and Co^2+^-substituted ZnFe_2_O_4_/ZnO laddered heterojunctions,^39–41^ wherein substitutional doping introduces intermediate energy states near the conduction band of ZnFe_2_O_4_. These novel electronic features promote the excitation of electrons from the O^2–^ 2p states in the valence band to higher-energy states (e.g., Zn^2+^ 4s orbitals), facilitating more efficient charge transfer and higher photocatalytic performance through laddered transitions. Importantly, this approach extends the photocatalytic response into the NIR region without the need for external scavengers, ensuring effective solar energy utilization. These laddered heterojunctions distinguish themselves from closely related ZnFe_2_O_4_/ZnO heterojunctions, which are typically type-II. Photogenerated electrons in the CB of ZnFe_2_O_4_ transfer to the CB of ZnO, while holes in the VB of ZnO transfer to the VB of ZnFe_2_O_4_. This spatially separates charges but at the cost of reducing the redox power of both electrons and holes (since both move to lower-energy levels).

Furthermore, the presence of a plasmonic Ag coating onto ZnO has been shown to enhance visible-to-NIR absorption through localized surface plasmon resonance, further boosting photocatalytic activity under solar irradiation.^40^ Fe^2+^-doped ZnFe_2_O_4_/ZnO core–shell nanoparticles in conjunction with CuO nanoparticles on their surface, forming a bijunctional photocatalyst with two distinct heterojunctions were used as a solar photocatalyst for scavenger free degradation of efficient degradation of textile dyes and antibiotics.^42^

These multifunctional nanoarchitectures thus demonstrate a synergistic approach to advancing solar-driven degradation of persistent organic dyes such as ALR. This study presents an efficient photocatalytic approach for the degradation of ALR utilizing a nanocomposite composed of Co^2+^-substituted zinc ferrite (CZFO) nanospheres embedded within zinc oxide (ZnO) nanosheets and surface-coated with plasmonic silver (Ag) nanoparticles. The engineered CZFO/ZnO heterojunction promotes effective separation of photogenerated charge carriers and extends light absorption into the visible and near-infrared (NIR) spectrum, substantially enhancing photocatalytic performance under solar irradiation. Under combined visible and NIR illumination, the nanocomposite achieved rapid and high-efficiency degradation of ALR, demonstrating its strong potential for scalable wastewater remediation. Comprehensive investigations under varied operational parameters were conducted to optimize degradation efficiency. Mechanistic insights obtained from scavenger studies identified hydroxyl (−OH) and superoxide (·O_2_^–^) radicals as the primary reactive species responsible for dye decomposition. Collectively, these results validate the CZFO/ZnO nanocomposite as a robust, sustainable photocatalyst for the removal of ALR from industrial effluents commonly generated by the food processing, beverage, textile, and cosmetic industries, which significantly contribute to dye-laden wastewater contamination.

## 2 Experimental Section

### 2.1 Materials

The following analytical-grade chemicals were used without further purification: iron­(III) nitrate nonahydrate (≥98%, Merck), zinc­(II) nitrate hexahydrate (≥96%, Merck), zinc­(II) acetate dihydrate (≥98%, Loba), cobalt­(II) nitrate hexahydrate (≥98%, Merck), sodium acetate trihydrate (≥98.5%, Merck), polyethylene glycol 1500 (PEG, Merck), sodium hydroxide (≥97%, Merck), ethylene glycol (EMPLURA, Merck), and silver nitrate (Merck) were used in the synthesis of the photocatalyst. Deionized water (18 MΩ•cm), Allura Red AC dye (80%, Loba), terephthalic acid (TA) (≥98%, Loba), and nitro blue tetrazolium chloride (NBT) (≥98%, Loba) were used in the photocatalytic studies. Potassium hydrogen phthalate (≥99.5%, Loba), sulfuric acid (≥98%, EMPLURA, Merck), potassium dichromate (≥99%, EMPLURA, Merck), mercury­(II) sulfate (≥98%, EMPARTA, Merck), and silver sulfate (≥98%, EMPARTA, Merck) were used to determine the chemical oxygen demand.

### 2.2 Synthesis of Co^2+^-Substituted ZnFe_2_O_4_ (CZFO) Nanospheres

The Co^2+^-substituted ZnFe_2_O_4_ (CZFO) were synthesized by the microwave-assisted solvothermal technique,^41^ 2.02 g (5 mmol) of Fe­(NO_3_)_3_.9H_2_O was dissolved in 20 mL ethylene glycol (EG) to form a red colored solution following the addition of Co­(NO_3_)_2_.6H_2_O (0.25 mmol) along with (2.5 – x) mmol of Zn­(NO_3_)_2_.6H_2_O, the resultant solution was named as solution A. Further, solution B was prepared by dissolving 6 g of sodium acetate trihydrate in 10 mL of EG. Solution C was prepared by dissolving 1 g of PEG-1500 in 10 mL of EG. All three solutions were added together and stirred vigorously for 30 min. The reaction mixture was then transferred to an 80 mL quartz vial and heated in a microwave synthesizer at 200 °C for 60 min. After synthesis, the resultant magnetic substance was collected by a permanent magnet and washed several times with water and ethanol. The samples were then kept for drying. The dried samples were then subjected to annealing at 400 °C for 4 h in the presence of atmospheric air.

### 2.3 Synthesis of CZFO/ZnO Nanocomposite by Microwave-Assisted Reflux Method

CZFO/ZnO composite was prepared in a mole ratio of CZFO relative to ZnO (1:12) by the microwave-assisted reflux method.^41^ For the synthesis, two solutions were prepared. Solution (a): zinc­(II) acetate dihydrate (3.4 g) was dissolved in 70 mL of DI water. In this solution, 300 mg of CZFO nanoparticles were dispersed. Solution (b): 2400 mg of NaOH was dissolved in DI water (30 mL). Solution (b) was added to the dispersion in solution (a) dropwise, followed by vigorous stirring for 20 min. The pH of the resultant mixture was maintained between 12 and 13. The final dispersion was transferred to a 500 mL round-bottom flask and exposed to microwaves for 30 min in a domestic microwave oven (LG, 800 W). After irradiation, the samples were centrifuged at 6000 rpm for 10 min and then dried in an oven at 80 °C.

### 2.4 Ag Coating on the Synthesized CZFO/ZnO Nanocomposite (Ag-CZFO/ZnO) by the Photoreduction Method

To deposit 2.5% silver on the surface of the synthesized nanocomposite,^43,44^ 395 mg of nanocomposite was dispersed in a solution containing 400 mL of DI water and 8 mg silver nitrate (AgNO_3_) and ultrasonicated for 6 min to ensure uniform dispersion. The resulting suspension was divided equally into four 500 mL glass beakers (100 mL in each) and exposed to direct sunlight for 2 h (12:00 p.m. to 2:00 p.m.) under clear sky conditions in the month of April at Manipal, India (GPS coordinates: 13.353046°N, 74.793905°E). After photoinduced deposition, the particles were recovered by filtration, washed thoroughly with DI water followed by ethanol, and dried at 80 °C for 12 h.

### 2.5 Characterizations

The phase purity and crystallographic structure of the synthesized materials were examined by powder X-ray diffraction (XRD) using a Rigaku Ultima IV diffractometer equipped with Cu Kα radiation (λ = 0.154 nm). XRD patterns were recorded in the 2θ range of 20 to 80° at a scan rate of 2°/min. Morphological analysis and microstructural features were investigated by using a field emission scanning electron microscope (FESEM, ZEISS ULTRA-55). The optical absorption characteristics of the as-prepared samples were evaluated by using a UV/Vis/NIR spectrophotometer (PerkinElmer Lambda 950) equipped with an integrating sphere in the diffuse reflectance mode. The reflectance data were converted into absorbance data using the Kubelka–Munk function. Tauc plots were used to estimate the optical band gap energies of the samples. X-ray Fluorescence (XRF) (Fischer Measurement Technologies (India) Pvt. Ltd., Goldscope SD 510) was used to analyze the amount of Ag deposited onto the photocatalyst.

### 2.6 Photoelectrochemical Studies

Photoelectrochemical (PEC) measurements of CZFO, CZFO/ZnO, and Ag-CZFO/ZnO electrodes were performed by using an electrochemical workstation (Autolab PGSTAT 204). Prior to film deposition, fluorine-doped tin oxide (FTO) substrates (1.0 × 1.5 cm^2^) were cleaned to research-grade standards.^45^ A suspension was prepared by dispersing 5 mg of the photocatalyst in 5 mL of ethanol containing dissolved PVDF as a binder. For electrical contacts, the area of the FTO substrate (0.5 cm^2^) was masked with Kapton tape. Subsequently, 15 μL of the nanoparticle suspension was drop-cast onto the substrate using a micropipette and dried for 30 s. This deposition procedure was repeated 12 times to obtain a uniform film. The prepared electrodes were then employed for electrochemical measurements in 0.5 M KOH aqueous electrolyte with a platinum (Pt) wire as a counter electrode and Ag/AgCl as a reference electrode. The charge separation efficiency was studied using electrochemical impedance spectroscopy (EIS) with a sinusoidal waveform in the frequency range of 10 kHz to 1 Hz. The photocurrent responses were recorded under a solar simulator (CEL-S500/350) equipped with an AM 1.5G filter, with an exposed electrode area of 1.0 cm^2^.

### 2.7 Photocatalytic Studies

Photocatalytic experiments were conducted to investigate the degradation of the ALR dye under artificial illumination. A 50 W white COB LED was used as the visible light source, and aluminum foil was employed to enhance the light reflection. For near-infrared (NIR) irradiation, a 1000 W halogen lamp was utilized. The concentrations of the dye and photocatalyst were set at 50 μM and 1 g/L, respectively. The photocatalyst was dispersed in 50 mL of the dye solution and kept in the dark for 1 h to establish an adsorption–desorption equilibrium. After equilibration, the suspension was exposed to artificial light. The visible light source (COB LED) was mounted on an air-cooled heat sink and positioned vertically inside a rectangular enclosure with the inner walls lined with aluminum foil to maximize light reflection. The NIR halogen lamp was placed 40 cm above the reaction solution. To manage heat from the light sources, a 10 mm thick water jacket was placed above the beaker containing the reaction solution.

Photodegradation was monitored by withdrawing 2 mL aliquots at designated time intervals. The samples were centrifuged to remove suspended photocatalyst particles, and the absorbance of the supernatant was measured by using a UV–Vis spectrophotometer (JASCO V650). The extent of dye degradation was assessed by tracking the change in absorbance, which was calculated using the following equation:

$$D\;(\%)=\frac{\left(C_{o}-C_{t}\right)}{C_{o}}\times 100\%$$

where *D* is the dye degradation percentage, *C*_0_ is the dye concentration (before light exposure), and *C_t_* is the dye concentration after an interval of time “*t*”.

After complete discoloration of the solution, the photocatalyst was recovered, dried, and reused in subsequent cycles to evaluate its reusability under identical conditions.

### 2.8 Confirmatory Test for Hydroxyl and Superoxide Radicals

The generation of hydroxyl radicals during the photocatalysis study was tested by using terephthalic acid as a probe.^46,47^ The photocatalyst (50 mg) was dispersed in a 50 mL solution of 166 mg of terephthalic acid and NaOH (0.05 M) in the presence of (50 μM) dye. The photocatalyst was kept for ultrasonication for 3 min to ensure better sample dispersion and kept in dark conditions for 1 h. Subsequently, the dispersion was exposed to light, and then 2 mL of the aliquot was collected after desired time interval and the photocatalyst in collected aliquot was separated out using centrifugation. The emission spectra of the solution before and after light exposure was recorded at excitation wavelength (λ_ex_) of 315 nm. The generation of superoxide radicals during the photocatalysis study was tested by using NBT solution as a probe.^48^ 50 mg of the photocatalyst was dispersed in 0.025 mM NBT solution in the presence of (50 μM) dye and was kept under dark condition for 1 h. Subsequently, the dispersion was exposed to light and 2 mL aliquot was collected after desired time interval. Further, after separation of the photocatalyst from the aliquot, the absorption spectra of the solution were recorded.

### 2.9 Effect of Photocatalyst Loading on the Rate of Dye Degradation

To evaluate the influence of photocatalyst loading on the dye degradation rate, experiments were conducted under the same conditions described in Section 2.6. The degradation of the dye (50 μM) was studied using varying photocatalyst concentrations: 0.5, 1, 2, 3, 4, and 5 g/L. Prior to irradiation, all reaction mixtures were subjected to dark conditions for 60 min to ensure adsorption–desorption equilibrium. The suspensions were then exposed to a combined light source (visible + NIR). At specified time intervals, aliquots were withdrawn, and the photocatalyst was separated by centrifugation. The absorption spectra of the resulting solutions were recorded to monitor dye degradation.

### 2.10 Effect of pH on the Dye Degradation Rate

The effect of pH on the photocatalytic degradation of the dye was studied by adjusting the pH of the dye solution (50 μM) to 5.0, 5.5, 6.0, 6.5, and 7.0 by using dilute NaOH or HCl. The photocatalyst was then added to achieve a concentration of 1 g/L. The suspensions were kept in the dark for 60 min to allow adsorption–desorption equilibrium, followed by irradiation under combined visible and NIR light. At predetermined time intervals, 2 mL aliquots were withdrawn and centrifuged to separate the photocatalyst, and the absorbance of the supernatant was recorded to assess dye degradation.

### 2.11 Effect of Electron Scavenging Agent on the Rate of Dye Degradation

The influence of an electron scavenger on the photocatalytic degradation of the dye was investigated by using hydrogen peroxide (H_2_O_2_) at a concentration of 1 mM. The photocatalyst (1 g/L) was dispersed in a 50 μM dye solution and kept in the dark for 60 min. After dark equilibration, H_2_O_2_ was added, and the suspension was irradiated using a combined visible and NIR light source. Aliquots (2 mL) were collected at selected time intervals, centrifuged to remove the photocatalyst, and the absorbance of the clear solution was measured to evaluate the degradation process.

### 2.12 Effect of Hole Scavenging Agent on the Rate of Dye Degradation

To assess the role of photogenerated holes in the dye degradation process, methanol (CH_3_OH) was introduced as a hole scavenger at a concentration of 2 mM. A suspension containing the photocatalyst (1 g/L) and dye (50 μM) was first equilibrated in the dark for 60 min. Methanol was then added to the system, and the mixture was irradiated using a combination of visible and NIR light. At predetermined time intervals, 2 mL samples were withdrawn and centrifuged to remove the photocatalyst, and the absorbance of the resulting supernatant was measured to evaluate the extent of degradation.

### 2.13 Chemical Oxygen Demand Measurements for the Pollutant Sample

The chemical oxygen demand (COD) of the dye solution was determined using the Closed Reflux Titrimetric Method in accordance with APHA Standard 5220 C. A 50 mL solution of Allura Red (50 μM) containing 1 g/L photocatalyst was prepared and kept in the dark for 60 min to attain adsorption equilibrium. The suspension was then exposed to combined visible and NIR light for 150 min. Following irradiation, the photocatalyst was separated by centrifugation, and a COD analysis was performed on the supernatant. For COD determination, 2.5 mL of the sample was refluxed with 1.5 mL of standard potassium dichromate solution and 3.5 mL of concentrated sulfuric acid containing silver sulfate as a catalyst in sealed COD digestion tubes. The mixture was heated at 150 °C for 2 h in a COD reactor. After the mixture cooled, the excess dichromate was titrated with standardized ferrous ammonium sulfate (FAS) solution using ferroin as an indicator. COD values were calculated based on the volume of FAS consumed. To validate the procedure and ensure accuracy, potassium hydrogen phthalate (KHP) with a theoretical COD of 300 mg/L was used as a standard. The recovery and percentage errors were calculated by comparing the measured COD value of KHP with its known theoretical value.

## 3 Results and Discussion

### 3.1 Structural Analysis

The crystallographic structure and phase composition of the synthesized nanomaterials CZFO, CZFO/ZnO, and Ag-CZFO/ZnO were analyzed by using X-ray diffraction (Figure 1). For the CZFO sample, distinct diffraction peaks at 2θ values corresponding to the (220), (311), (222), (400), (422), (511), (440), and (533) planes are observed. These match well with the cubic spinel structure of ZnFe_2_O_4_ (JCPDS no. 01–1109), confirming the formation of the spinel ferrite phase. The incorporation of Co^2+^ into the ZnFe_2_O_4_ lattice does not result in distinct new peaks due to its low doping concentration. However, a slight shift of approximately 0.07° in the diffraction peaks compared to pure ZnFe_2_O_4_ indicates successful substitution of Co^2+^ ions into the spinel structure, with no evidence of secondary phase formation.^41^ In the CZFO/ZnO composite, additional diffraction peaks appear at 2θ values of approximately 31.8, 34.5, 36.3, 47.6, 56.6, 62.9, and 68.0°, which correspond to the (100), (002), (101), (102), (110), (103), and (112) planes of hexagonal wurtzite ZnO (JCPDS no. 05–0664). These peaks confirm the successful incorporation of ZnO into the composite without affecting the spinel CZFO phase, as no peak splitting or significant shifts are observed. Upon silver coating, the XRD pattern of the Ag-CZFO/ZnO sample shows the same characteristic peaks of CZFO and ZnO along with a few additional weak intensity peaks around 38.1, 44.3, 64.4, and 77.4°, corresponding to the (111), (200), (220), and (311) planes of face-centered-cubic metallic silver (Ag), in agreement with JCPDS no. 65–2871. These peaks confirm the presence of crystalline Ag on the surface of the CZFO/ZnO composite.^40^ No extraneous peaks related to impurities or secondary phases were detected in any of the samples, indicating the high phase purity of the synthesized materials. The successful formation of the ternary Ag-CZFO/ZnO nanocomposite with well-defined crystalline phases is thus confirmed by XRD analysis.

**Figure 1:**
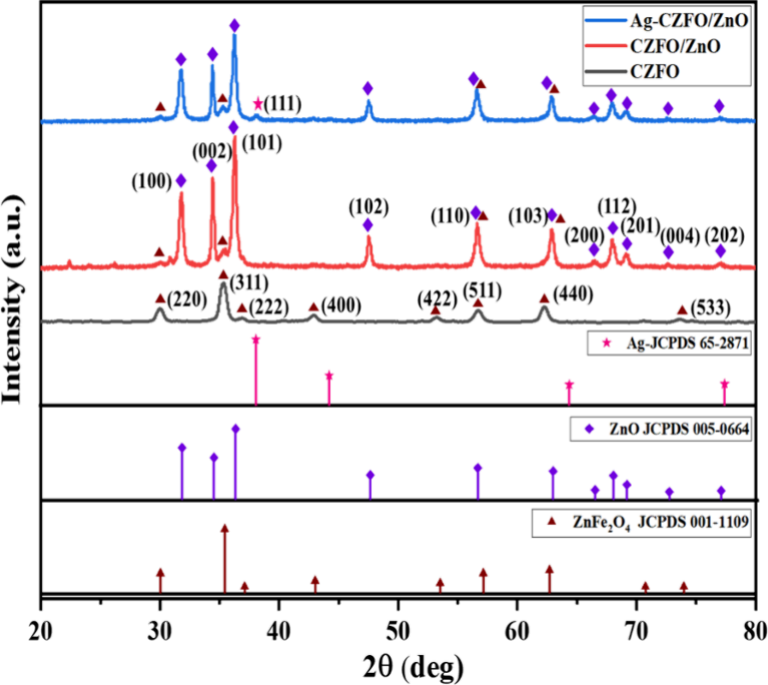
X-ray diffraction patterns of CZFO, CZFO/ZnO (annealed at 350 °C), and the Ag-CZFO/ZnO nanocomposite sample.

The optical properties of the Ag-CZFO/ZnO nanocomposite were investigated by using UV–Vis–NIR spectroscopy, and the results are shown in Figure 2. The absorbance spectrum (Figure 2a) reveals a broad absorption profile extending from the ultraviolet to the near-infrared region with a distinct absorption edge around 925 nm. This significant red shift is attributed to the localized surface plasmon resonance (LSPR) effect of Ag nanoparticles on the composite surface, enhancing light absorption into the NIR region. The optical band gap of the material was estimated using the Kubelka–Munk function plotted against photon energy (hν), assuming an indirect allowed transition. As shown in Figure 2b, the extrapolated linear portion of the plot yields a band gap of approximately 1.45 eV, which is notably lower than that of pristine ZnFe_2_O_4_ or ZnO. The narrowed band gap and extended light absorption range suggest that the Ag-CZFO/ZnO nanocomposite is well-suited for photocatalytic applications under broad-spectrum solar irradiation.

**Figure 2:**
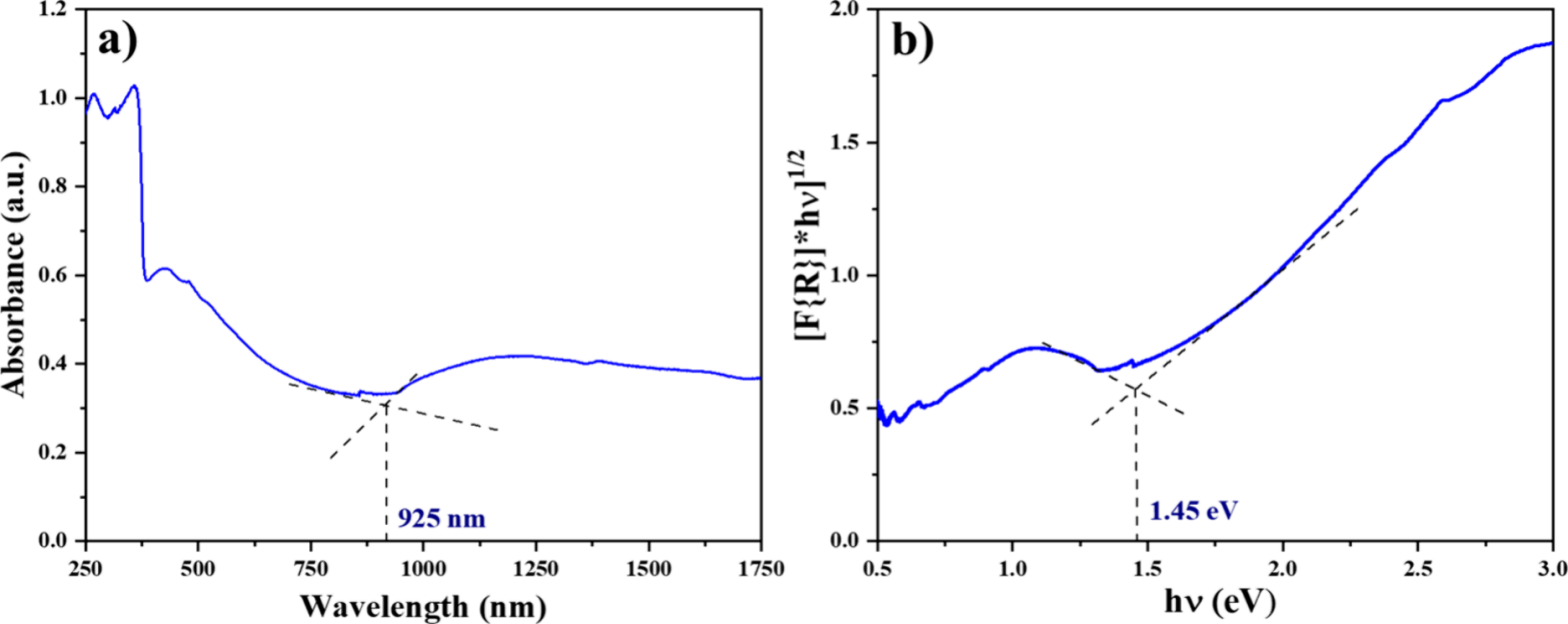
(a) UV–Vis–NIR absorbance spectrum of the Ag-CZFO/ZnO nanocomposite. (b) Indirect band gap determination from the Kubelka–Munk plot using the diffused reflectance spectrum.

Field emission scanning electron microscopy (FESEM) images of the Ag-CZFO/ZnO nanocomposite are presented in Figure 3 at varying magnifications (10, 40, and 90 KX). The low-magnification image (Figure 3a) reveals a densely packed sheet-like morphology with distinct surface texture. At higher magnifications (Figure 3b,c), the nanosheets appear to have sharp edges and well-defined layered structures.

**Figure 3:**
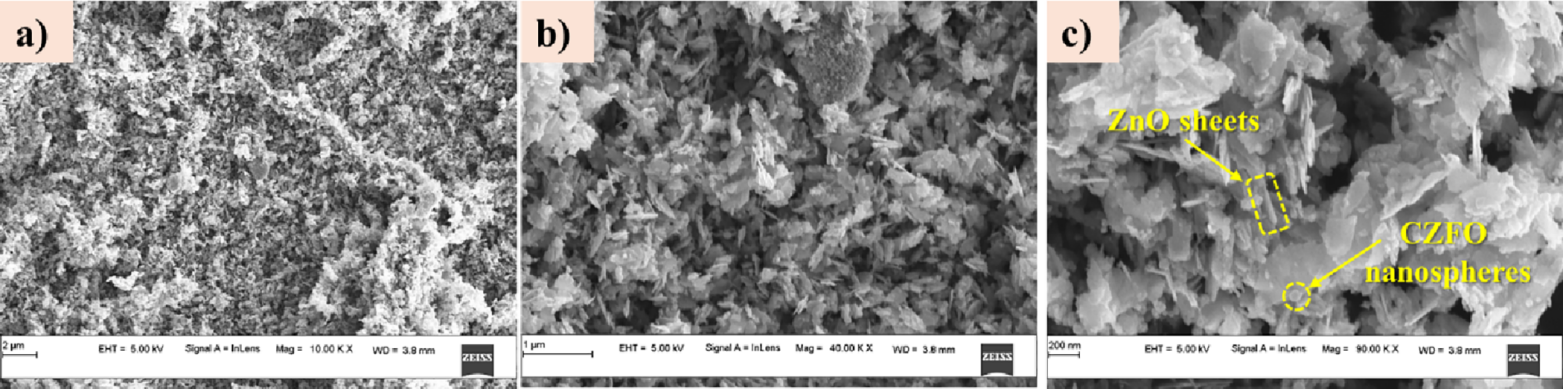
FESEM images of the Ag-CZFO/ZnO nanocomposite at (a) 10, (b) 40, and (c) 90 KX magnifications. CZFO nanoparticles are visibly distributed over the surface of ZnO nanosheets.

Notably, CZFO nanoparticles with sizes ranging from 20 to 40 nm are clearly observed anchored on the surface of the ZnO sheets. The sheets themselves exhibit a thickness of approximately 30–40 nm, while the lateral dimensions range from 100 to 400 nm. The intimate contact between the CZFO nanoparticles and ZnO sheets is expected to promote efficient charge separation and transfer, which is advantageous for photocatalytic applications.

Uniform and homogeneous distributions of all constituent elements were observed through elemental analysis (EDS) and corresponding elemental maps. The Zn and O signals reveal the widespread presence of ZnO nanosheets, while Fe and Co are evenly distributed throughout the matrix, indicating the successful incorporation of Co^2+^ ions into the ZnFe_2_O_4_ lattice without phase segregation. To verify the elemental constitution and spatial distribution within the Ag-CZFO/ZnO nanocomposite, energy-dispersive X-ray spectroscopy (EDS) coupled with elemental mapping was performed. The EDS spectrum confirms the presence of Zn, Fe, Co, O, and Ag, validating the successful synthesis of CZFO embedded in ZnO nanosheets with surface modification by Ag nanoparticles. A minor carbon signal is also detected, which originates from the carbon substrate used during sample preparation.^40^

The Ag mapping further confirms the uniform dispersion of metallic Ag, which is expected to contribute significantly to the localized surface plasmon resonance (LSPR) effect, enhancing visible- to near-infrared light absorption and facilitating charge separation. X-ray Fluorescence (XRF) was used to analyze the amount of Ag deposited onto the photocatalyst, and 1.95% of Ag deposited on our sample was confirmed, closely matching our intended value of 2.5%.

Brunauer–Emmett–Teller (BET) surface area analysis was performed to evaluate the textural properties of the synthesized materials.^40^ Following the Ag coating on the CZFO/ZnO nanocomposite, a slight reduction in surface characteristics was observed. The specific surface area decreased marginally from 35 m^2^g^–1^ to 34 m^2^g^–1^. Similarly, the average pore diameter reduced from 35 to 34 nm, while the total pore volume decreased from 0.30 cm^3^g^–1^ to 0.28 cm^3^g^–1^. These minor reductions suggest partial pore blockage or surface coverage by the Ag.

### 3.2 Adsorption Studies

To ensure accurate evaluation of photocatalytic performance, establishing adsorption–desorption equilibrium prior to light irradiation is essential. The adsorption behavior of ALR on the Ag-CZFO/ZnO nanocomposite was investigated under dark conditions. It was observed that equilibrium between adsorption and desorption was attained within 60 min; 23% of dye was adsorbed in 60 min and was constant beyond this time (Figure 4). Accordingly, all photocatalytic degradation experiments were initiated only after 1 h of dark stirring to ensure equilibrium conditions.

**Figure 4:**
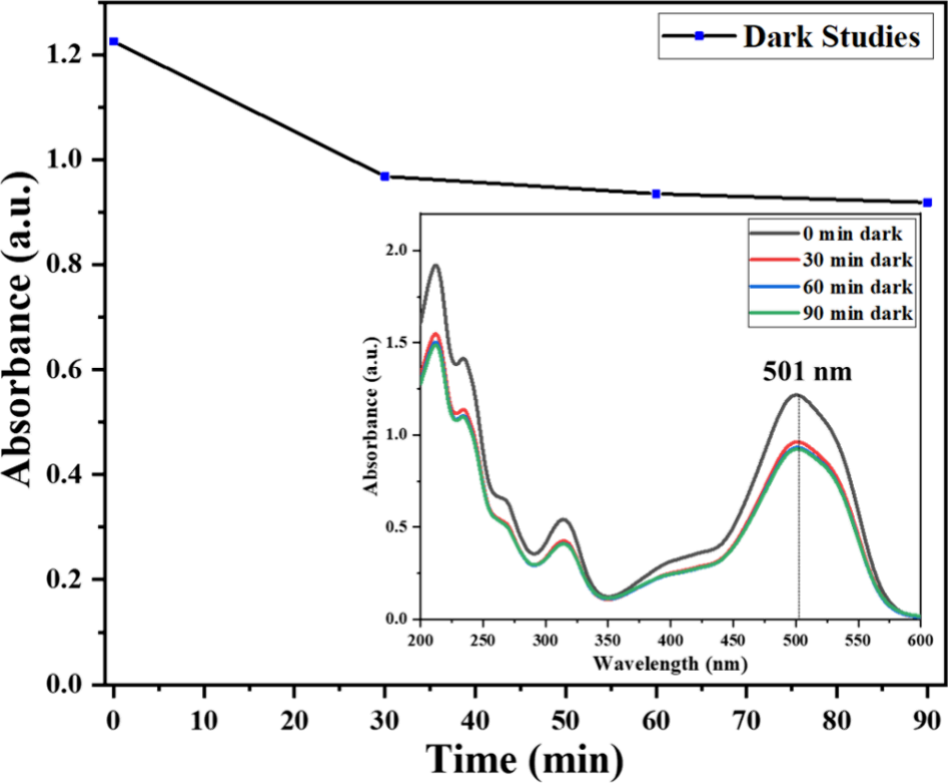
Variation of ALR dye concentration with time during adsorption (in the absence of light) with Ag-CZFO/ZnO nanocomposite, and the inset shows the corresponding change in the absorbance spectra of ALR dye with respect to time.

Figure 4 illustrates the temporal variation in the ALR dye concentration during the dark adsorption phase, highlighting the stabilization trend. The inset depicts the corresponding UV–Vis absorbance spectra of ALR dye at various time intervals, confirming the attainment of equilibrium.

### 3.3 Photodegradation Studies

After establishing the adsorption–desorption equilibrium, we evaluated the photocatalytic activity of the nanocomposite under both visible and near-infrared (NIR) light irradiation. For visible light, we used a 50 W white COB LED, while a 1000 W halogen lamp was employed to provide NIR illumination. The ALR dye solution, which contained the photocatalyst, was exposed to these light sources, and we monitored the degradation process spectroscopically. The photocatalytic degradation behavior of ALR dye in the presence of (i) Ag-CZFO/ZnO nanocomposite (ii) CZFO/ZnO and (iii) CZFO is illustrated in the Figure 5a. Using Ag-CZFO/ZnO nanocomposite, during the initial dark phase (−60 to 0 min), a moderate decrease in dye concentration was observed, indicating adsorption of the dye onto the nanocomposite surface and the establishment of adsorption–desorption equilibrium within 60 min. Upon exposure to light, using a 50 W white COB LED and a 1000 W halogen lamp, a sharp decline in the dye concentration was recorded, with approximately 96% degradation achieved within 150 min. The corresponding spectral changes and color changes during the photodegradation of ALR are shown in Figure 5b. A consistent reduction in the intensity of the characteristic peak at 501 nm, with no new peaks appearing, suggests the efficient degradation of the dye chromophore without formation of light-absorbing intermediates. It can be noted that the degradation using CZFO was much slower than CZFO/ZnO, inferring that the heterojunction favors charge separation. Comparing the degradation curve of CZFO/ZnO with that of Ag-CZFO/ZnO, we can observe the highest degradation rate for the Ag-coated sample, attesting to the fact that the plasmonic-coated sample further enhances the charge separation. More details are discussed in the mechanism section at the end of the manuscript. Together, the kinetic and spectral data clearly demonstrate the high photocatalytic efficiency of the synthesized nanocomposite under visible and NIR light irradiation. These results conclusively establish the Ag-CZFO/ZnO nanocomposite as a highly efficient visible-light-driven photocatalyst for the degradation of organic dye pollutants.

**Figure 5:**
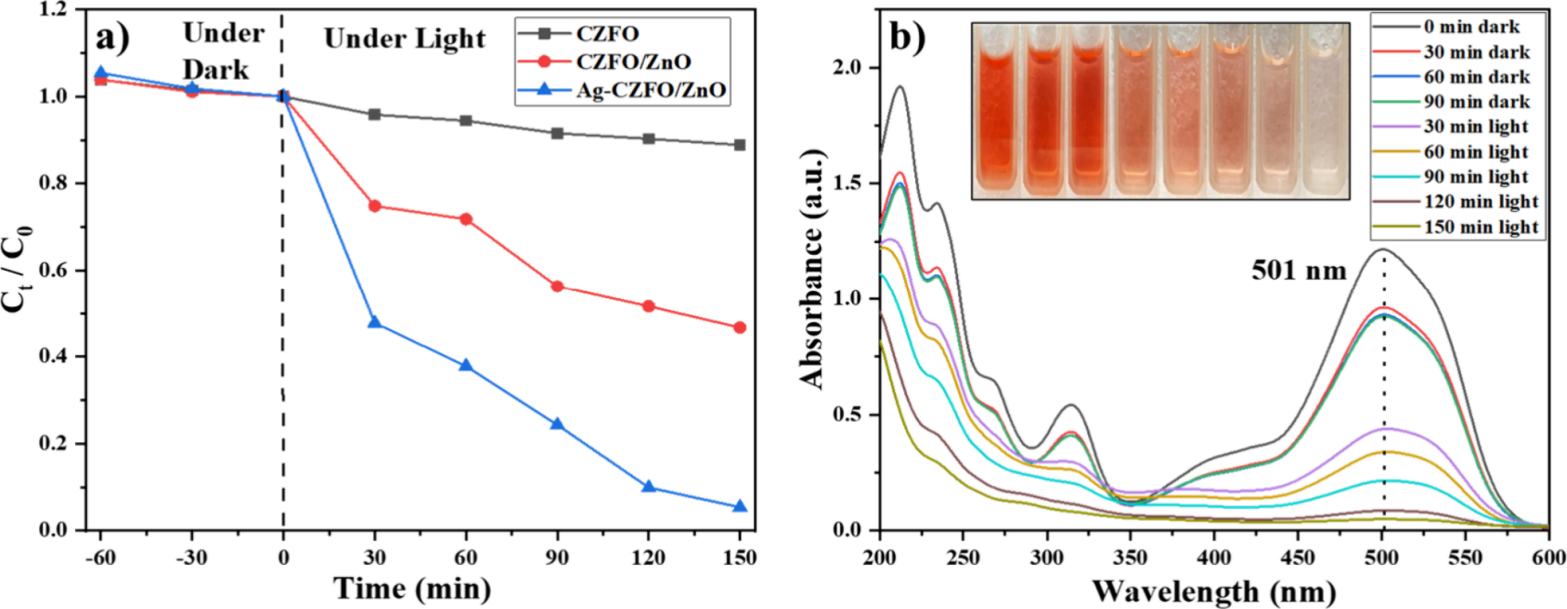
(a) Relative concentration of ALR dye versus light exposure time with CZFO, CZFO/ZnO, and Ag-CZFO/ZnO nanocomposite. (b) Absorption spectra of ALR dye solution after various light exposure durations in the presence of the Ag-CZFO/ZnO nanocomposite; inset: visual color degradation over time.

### 3.4 EIS Studies for Charge Carrier Dynamics

The charge transfer resistance (*R*_ct_) was evaluated from electrochemical impedance spectroscopy (EIS), which offers qualitative insights into the charge transport processes occurring across the photoelectrode/electrolyte interface. The corresponding Nyquist plots are shown in Figure 6. It is observed that the bare CZFO electrode exhibits a large semicircle, indicating high charge transfer resistance and poor interfacial charge transport. Upon forming a heterojunction between CZFO and ZnO, the semicircle diameter decreases significantly, reflecting reduced *R*_ct_ value and more efficient charge separation. Notably, Ag-CZFO/ZnO shows the smallest semicircle among all samples, confirming the lowest interfacial resistance and the fastest charge transfer process at the electrode/electrolyte interface. The incorporation of conformal Ag coating not only improves conductivity but also enhances charge carrier dynamics by facilitating electron transport and suppressing recombination. These results are consistent with the photocurrent measurements, demonstrating that Ag-CZFO/ZnO possesses superior photoelectrochemical performance compared to the individual components and the binary heterojunction.

**Figure 6:**
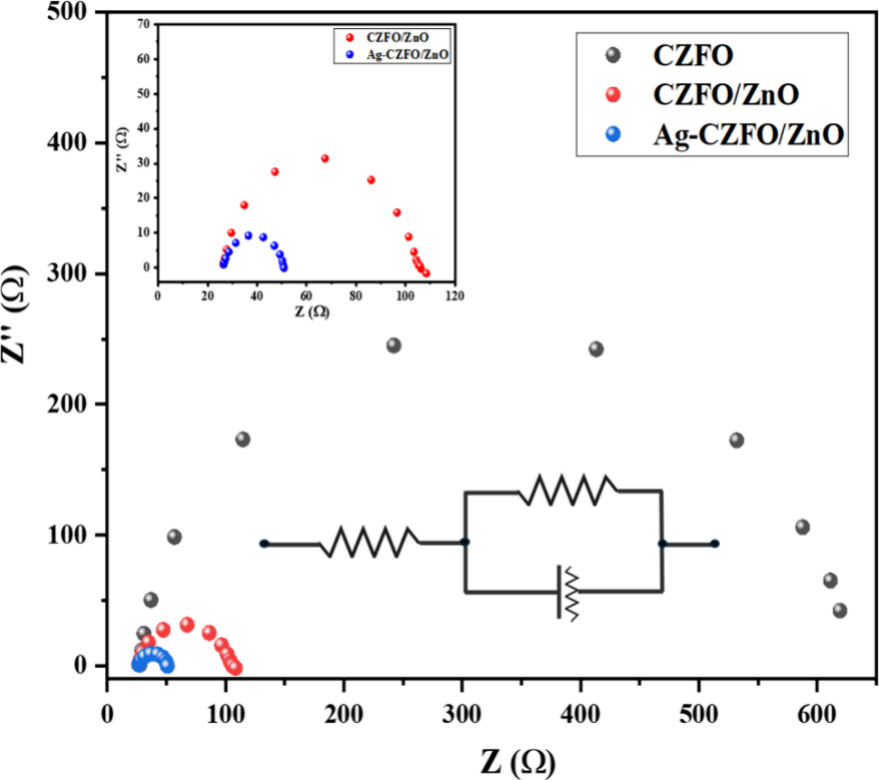
Nyquist plot of CZFO, CZFO/ZnO, and Ag-CZFO/ZnO photocatalysts; inset showing equivalent circuit model and the zoomed Nyquist plot of CZFO and CZFO/ZnO only.

### 3.5 Effect of Photocatalyst Loading on the Photocatalytic Dye Degradation

The effect of varying photocatalyst loading on the degradation efficiency of the ALR dye was systematically investigated using nanocomposite concentrations ranging from 0.5 to 5 g/L (Figure 7). photocatalytic degradation was minimal at a concentration of 0.5 g/L, achieving only 78% removal after 150 min of light exposure. However, increasing the photocatalyst dosage to 1 g/L significantly improved degradation to 96%, indicating a greater availability of active sites for reactive oxygen species (ROS) generation. Higher catalyst loading (up to 5 g/L) resulted in only marginal improvements, with degradation rates of 96.5%, 99.7%, 99.9%, and ultimately 100%, respectively. This limited enhancement is likely due to the saturation of surface-active sites and increased turbidity, which restricts light penetration at higher dosages.

**Figure 7:**
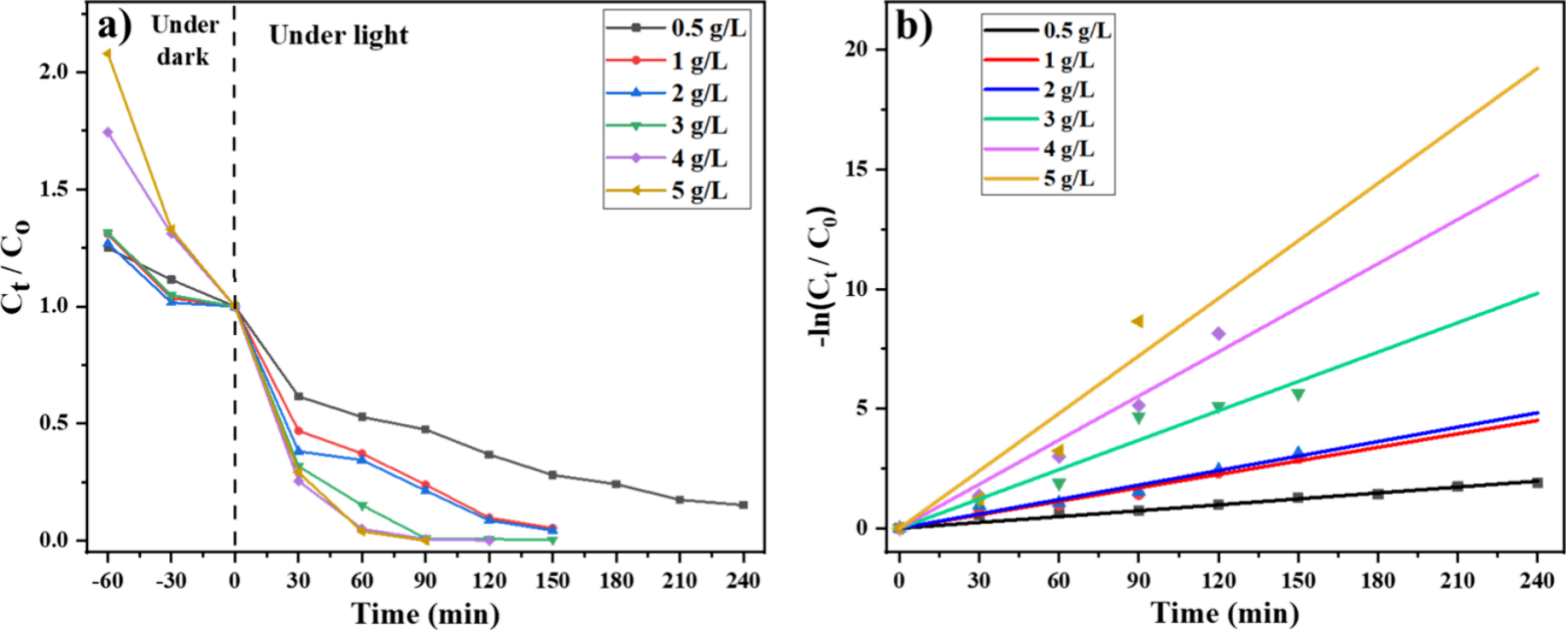
(a) Photocatalytic degradation kinetics of ALR dye in the presence of different concentrations (0.5 1, 2, 3, 4, and 5 g/L) of Ag-CZFO/ZnO nanocomposite and (b) their respective first-order kinetic plots.

This trend is further supported by the pseudo-first-order kinetic plots shown in the right panel. The slope of each linear fit represents the apparent rate constant (*k*). A sharp increase in the rate constant was observed when increasing the catalyst from 0.5 (0.0082 min^–1^) to 1 g/L (0.0188 min^–1^), while further increments resulted in diminishing returns: 0.0201 (2 g/L), 0.0409 (3 g/L), 0.0614 (4 g/L), and 0.0801 min^–1^ (5 g/L). These results suggest that while higher catalyst loadings provide more active sites, excessive amounts can hinder effective photon absorption due to light scattering and particle agglomeration. Thus, 1 g/L was determined to be the optimal photocatalyst loading for efficient degradation and was used in all subsequent experiments.

### 3.6 Effect of pH on the Photocatalytic Dye Degradation

The efficiency of photocatalytic dye degradation is closely related to how dye molecules adsorb onto the surface of the photocatalyst, which is significantly influenced by the pH of the reaction medium. The surface charge of the photocatalyst and the ionization state of the dye vary with pH, affecting the electrostatic interactions that control both the adsorption and subsequent degradation. To identify the optimal pH for photocatalytic activity, degradation experiments were conducted at various pH levels ranging from 5.0 to 7.0 (Figure 8).

**Figure 8:**
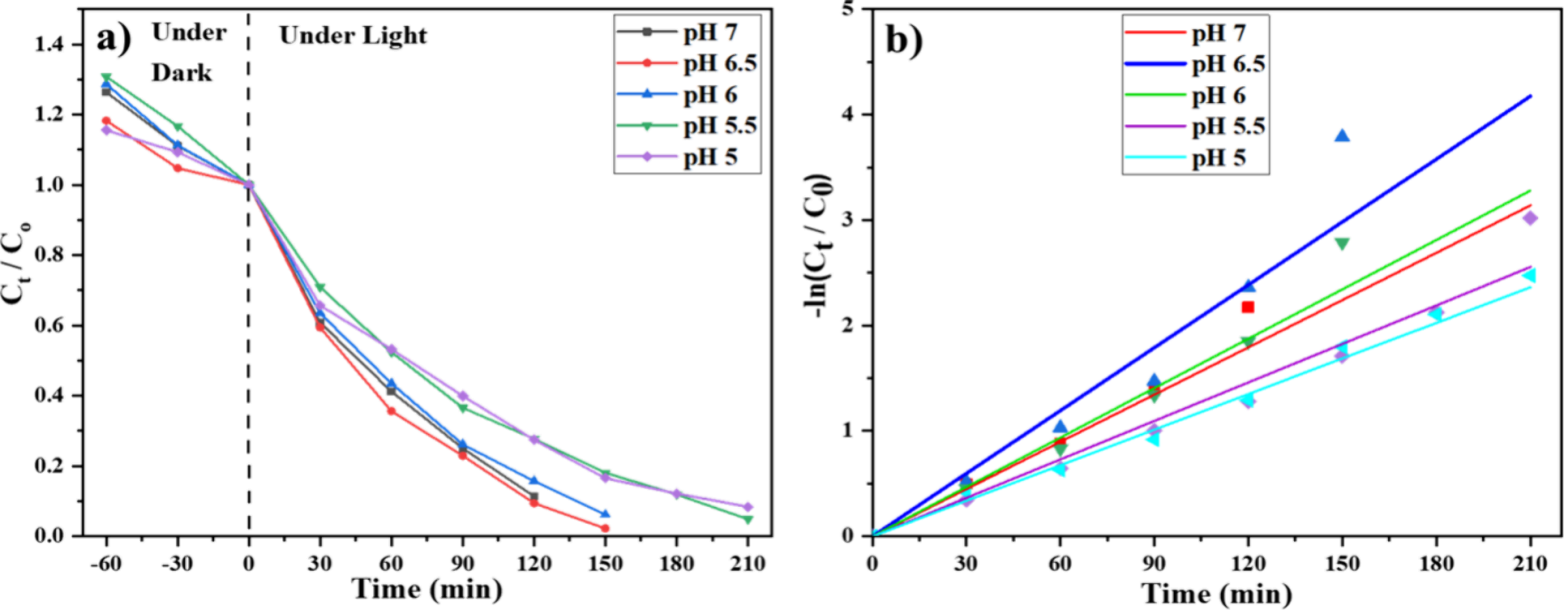
(a) Variation of ALR concentration with time and varying pH under combined visible and NIR light irradiation in the presence of the Ag-CZFO/ZnO nanocomposite and (b) the respective first-order kinetic plots.

As shown in the kinetic analysis, the degradation rate increased with pH up to a maximum at pH 6.5, which yielded the highest apparent rate constant of 0.01988 min^–1^. At slightly lower or higher pH values, a gradual decline in the rate constants was observed: 0.01562 min^–1^ at pH 6.0, 0.01494 min^–1^ at pH 7.0, 0.01217 min^–1^ at pH 5.5, and 0.01125 min^–1^ at pH 5.0. These results indicate that the photocatalyst exhibits optimal surface properties for dye adsorption and ROS-mediated degradation near neutral pH, with the pH range of 6.5–7.0 being most favorable for efficient photocatalysis. This optimal range likely corresponds to a balance between sufficient surface charge and minimal dye repulsion, facilitating effective interaction between the dye and catalyst surface.

### 3.7 Effect of Electron Scavenging Agent on the Rate of Photocatalytic Dye Degradation

The effect of an electron scavenging agent on the photocatalytic degradation of the ALR dye was investigated by adding hydrogen peroxide (H_2_O_2_) at a concentration of 1 mM to the reaction system (Figure 9). The introduction of H_2_O_2_ significantly improved the degradation efficiency, resulting in complete dye removal within 90 min of light exposure. The calculated pseudo-first-order rate constant was 0.0476 min^–1^, which is more than double the rate observed without the scavenger (0.0188 min^–1^).

**Figure 9:**
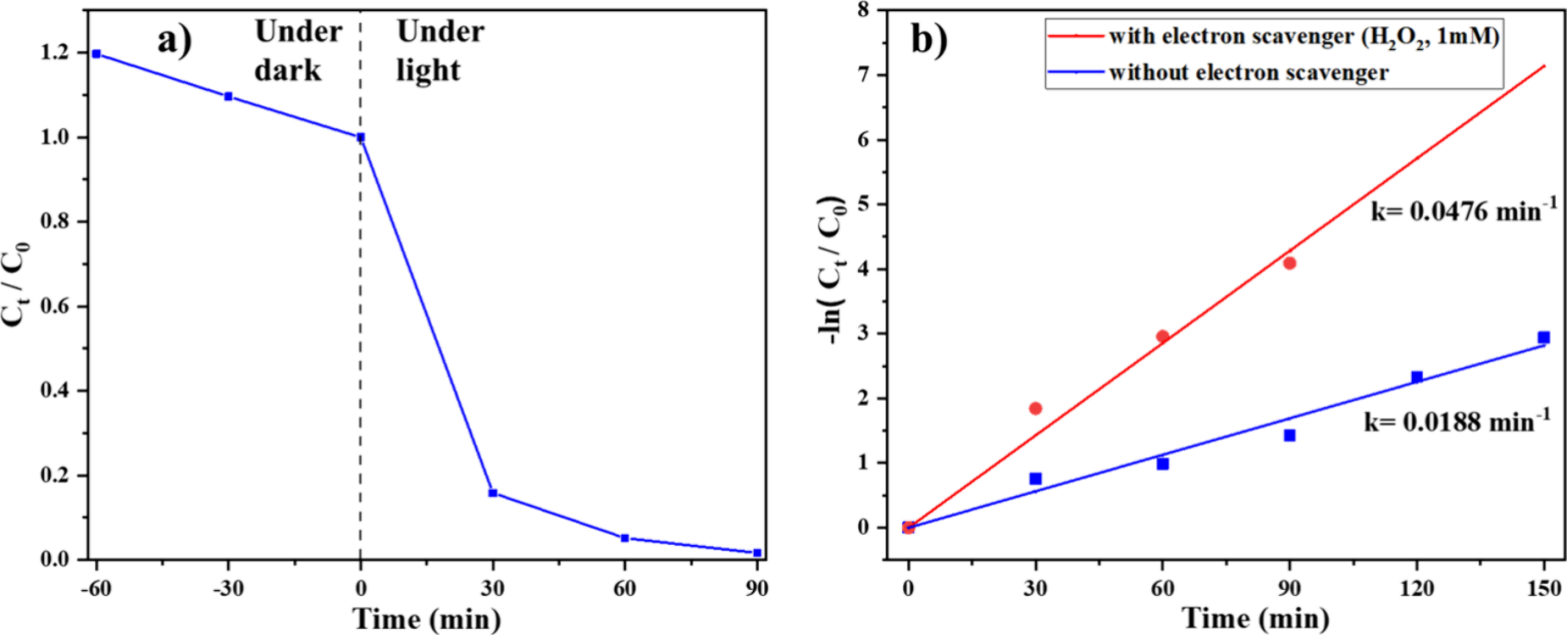
(a) Relative concentration of ALR dye with respect to the duration of exposure to light in the presence of Ag-CZFO/ZnO nanocomposite and in the presence of an electron scavenging agent. (b) Their respective first-order kinetic plots in comparison to dye degradation without the addition of a scavenging agent.

This substantial increase in the degradation rate can be attributed to H_2_O_2_’s ability to effectively capture photogenerated electrons, thereby reducing electron–hole recombination. The decrease in recombination enhances the availability of photogenerated holes, which can directly oxidize dye molecules or indirectly contribute to degradation through the formation of ROS, such as hydroxyl radicals (•OH) and superoxide radicals (•O_2_^–^). Thus, the addition of an electron scavenger not only accelerates the degradation kinetics but also promotes the complete mineralization of the dye through both direct and indirect oxidative processes.

### 3.8 Effect of Hole Scavenging Agent on the Rate of Photocatalytic Dye Degradation

To evaluate the role of photogenerated holes in the degradation process, methanol (CH_3_OH) was introduced as a hole scavenger at a concentration of 2 mM. The presence of CH_3_OH resulted in a significant decrease in photocatalytic efficiency (Figure 10). Complete degradation of ALR dye required 210 min of light exposure, and the rate constant was reduced to 0.01541 min^–1^, compared to 0.0188 min^–1^ in the absence of the hole scavenger, where degradation was achieved within 150 min.

**Figure 10:**
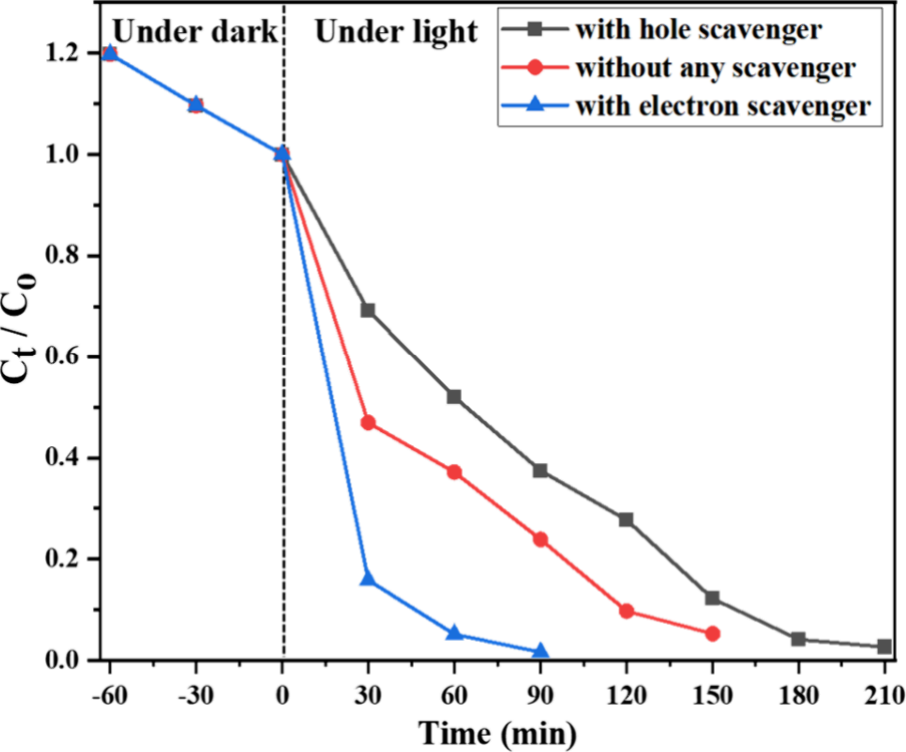
Progress of photocatalytic degradation of ALR dye with and without the presence of scavengers (hole and electron scavenger).

This decrease in the degradation rate indicates that methanol effectively interacts with the photogenerated holes, reducing their availability for dye oxidation. Consequently, both the direct oxidation pathway and the formation of secondary reactive oxygen species, such as hydroxyl radicals, are suppressed. These observations confirm that photogenerated holes play a crucial role in the photocatalytic degradation of the dye, significantly contributing to its mineralization either directly or through the generation of potent oxidizing species.

### 3.9 COD-Based Validation of Photocatalytic Efficiency

To assess the photocatalytic efficiency of the Ag-CZFO/ZnO nanocomposite, a COD analysis was performed using ALR dye solution as a model organic pollutant. COD measures the amount of oxygen needed to chemically oxidize organic matter, making it a critical parameter for evaluating water quality. The measurements were carried out using the standard closed reflux titrimetric method following the guidelines of APHA 5220 C. The photocatalytic activity of the Ag-CZFO/ZnO nanocomposite was evaluated by comparing the COD values before and after light exposure. The initial COD of the dye solution was 31.0 ppm, which decreased to below 5 ppm after treatment with an estimated measurement error of ± 2%. This significant reduction in COD indicates substantial mineralization of the dye molecules, confirming the high photocatalytic efficiency of the synthesized nanocomposite for potential wastewater treatment applications.

### 3.10 Reusability and Stability of the Photocatalyst

For effective wastewater treatment applications, a photocatalyst must exhibit good reusability and stable performance over multiple cycles without causing secondary pollution. To evaluate the durability of the synthesized Ag-CZFO/ZnO nanocomposite, recycling experiments were conducted over five consecutive cycles of photodegradation using the ALR dye as the model pollutant. After each cycle, the photocatalyst was recovered through centrifugation, thoroughly washed, and then redispersed in a fresh dye solution for the next cycle.

As illustrated in Figure 11a, the nanocomposite maintained a consistent degradation profile across all five cycles with only a marginal decline in efficiency. The corresponding degradation percentages (Figure 11b) were 95.95, 95.8, 94.82, 92.91, and 92.24% from cycle 1 to cycle 5, respectively. This minimal decrease in activity suggests that the photocatalyst retains its structural integrity and active sites throughout repeated use. We have verified the integrity of the photocatalyst after 5 reuses through the XRD patterns. There is no difference in the XRD patterns before and after reuse, suggesting the intactness of our photocatalyst (Figure 11c). Overall, the results confirm the excellent reusability and stability of the nanocomposite, supporting its potential for cost-effective and sustainable photocatalytic wastewater treatment.

**Figure 11:**
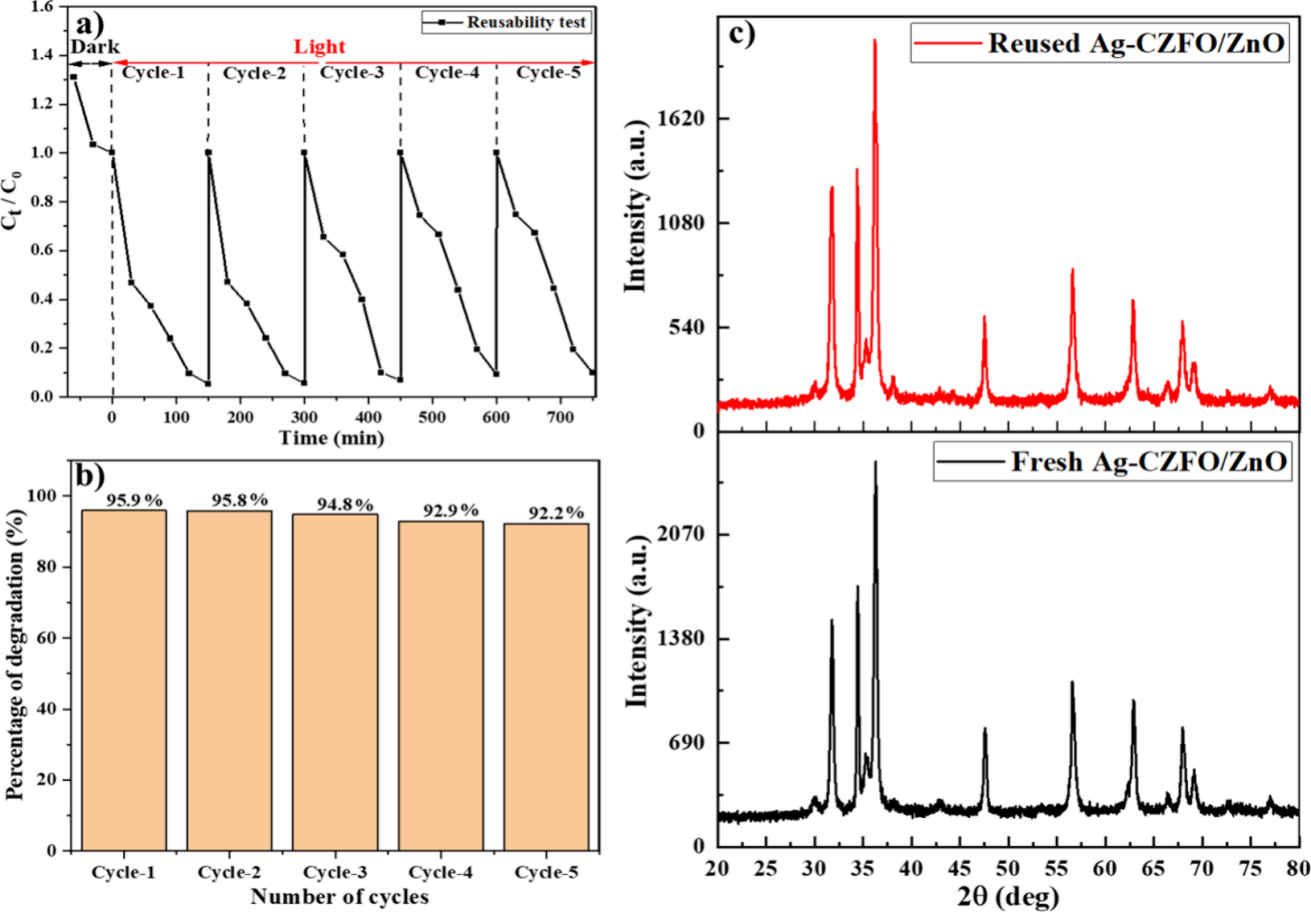
(a) Photodegradation of ALR using recycled photocatalyst for 5 reuse cycles, (b) percentage of dye degradation over different reuse cycles, and (c) XRD patterns of Ag-CZFO/ZnO nanocomposite before and after 5 successive reuse cycles.

### 3.11 Detection of Reactive Oxygen Species (ROS)

The involvement of ROS, such as hydroxyl radicals (•OH) and superoxide radicals (•O_2_^–^), in the photocatalytic degradation process was confirmed using specific probe molecules.^49^ To investigate the generation of hydroxyl radicals, terephthalic acid was used as a fluorescent probe. This probe selectively reacts with •OH to form 2-hydroxyterephthalic acid, which exhibits a strong photoluminescence emission at around 425 nm. The photoluminescence (PL) intensity increased significantly after 20 and 40 min of light exposure in the presence of the nanocomposite, indicating substantial generation of hydroxyl radicals (Figure 12a). In contrast, samples kept in the dark showed negligible emission, confirming that •OH radicals were formed due to light exposure.

**Figure 12:**
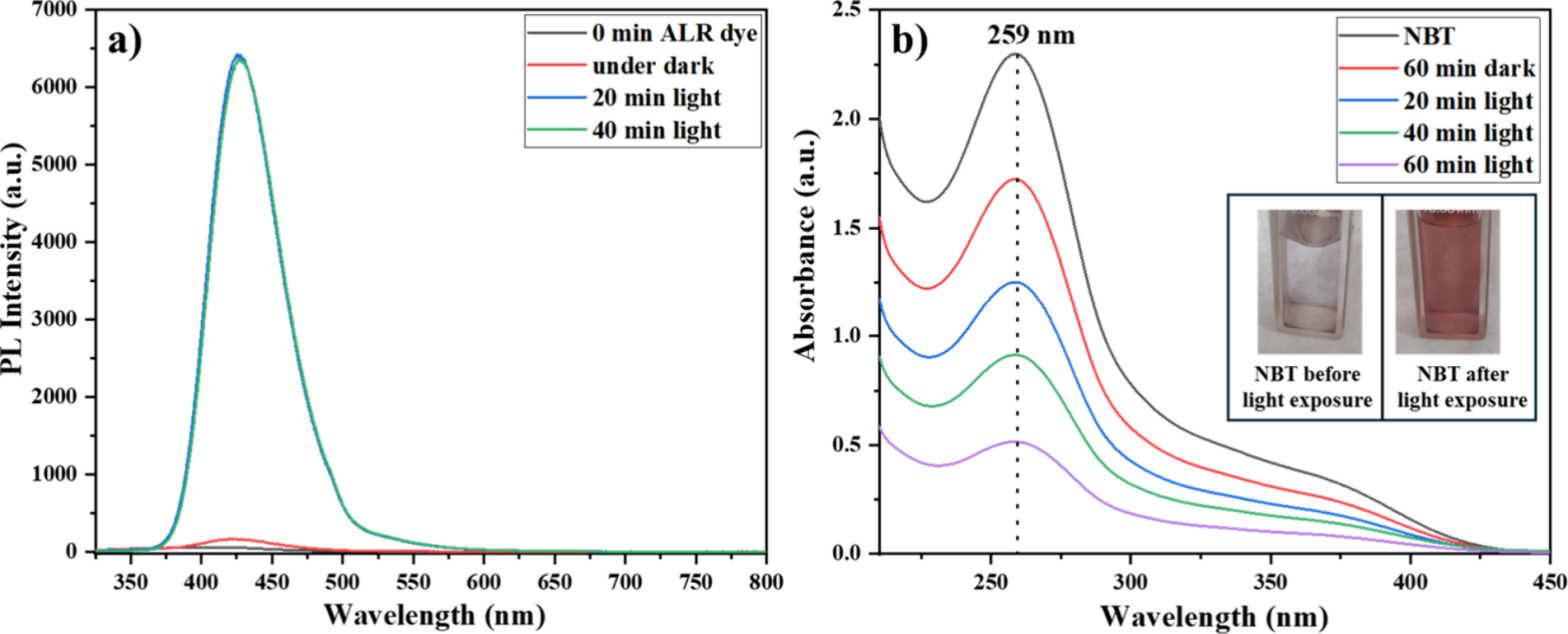
Detection of reactive oxygen species. (a) Hydroxyl radicals: emission spectra of terephthalic acid solution before and after light exposure with Ag-CZFO/ZnO nanocomposite and (b) superoxide radicals: absorption spectra of NBT solution after 60 min in the dark and under illumination; inset: visual change in NBT solution postillumination.

The formation of superoxide radicals was assessed using nitro blue tetrazolium (NBT), which undergoes reduction in the presence of •O_2_^–^, resulting in a blue-colored formazan. Figure 12b displays the UV–vis absorbance spectra of NBT before and after light exposure. A remarkable decrease in the characteristic absorption peak at 259 nm was observed with increasing light exposure times of 20, 40, and 60 min, indicating the consumption of NBT due to its reaction with the photogenerated superoxide radicals. The inset image further supports this finding as it shows a visible color change from colorless to blue, confirming formazan formation.

These findings clearly demonstrate the generation of both hydroxyl and superoxide radicals during the photocatalysis process, validating their essential role in the effective degradation of Allura Red AC dye by using the synthesized nanocomposite photocatalyst.

### 3.12 Photocurrent Measurements

The photoresponse of the photocatalysts was evaluated using chronoamperometry at an applied bias of 0.45 V vs Ag/AgCl for 200 s with a 20 s light on–off cycle. CZFO/ZnO exhibited a 10-fold improvement compared to CZFO, while the photocurrent density of Ag-CZFO/ZnO was nearly 100 times higher than that of bare CZFO (Figure 13). These results indicate that bare CZFO is less effective in separating the photogenerated charge carriers required for photocurrent generation. The formation of the CZFO/ZnO heterojunction significantly suppresses charge recombination, thereby enhancing charge separation and transport under illumination. Furthermore, Ag coating on the CZFO/ZnO heterostructure results in a pronounced enhancement of the photocurrent response, which can be attributed to plasmon-induced charge transfer and improved interfacial kinetics, which is an essential requirement for photocatalytic degradation.

**Figure 13:**
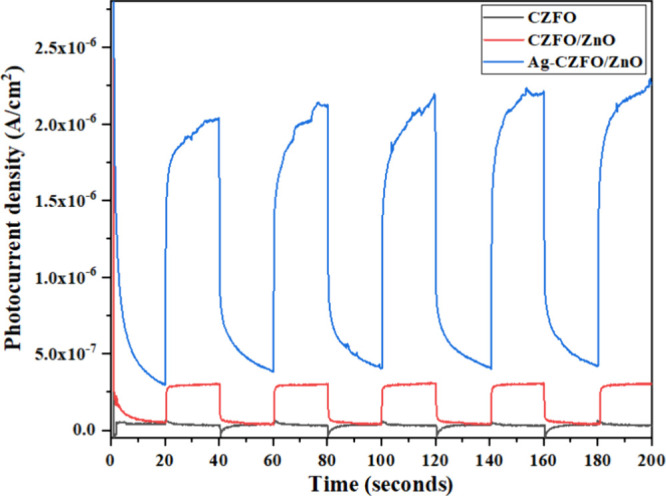
Chronoamperometric measurements to study the generation of photocurrent using CZFO, CZFO/ZnO, and Ag-CZFO/ZnO photocatalysts with applied bias of 0.45 V vs Ag/AgCl. Solar simulator was used with 20 s on–off cycles.

### 3.13 Mechanism of Photocatalytic Degradation Using Ag-CZFO/ZnO Photocatalyst

Band potentials of different components play an important role in a heterojunction photocatalyst; they help to understand the type of heterojunction and therefore the photocatalytic mechanism. Previously, the band potentials were identified using X-ray photoelectron spectroscopy.^41^ CZFO and ZnO form a type-1 heterojunction in view of the favorable positioning of their conduction band minima (CBM) and valence band maxima (VBM). Interestingly, Co^2+^ substitution forms a laddered type-1 heterojunction between CZFO and ZnO with reduced charge recombination (Figure 14). Laddered transitions help the photogenerated electrons reach the Zn^2+^ level, which is poised to transfer the electrons to ZnO by utilizing NIR light. The performance was further enhanced when Ag was selectively coated on the heterojunction, enabling better charge separation as electrons flow from ZnO to Ag, forming a Schottky junction. Owing to the LSPR behavior of the Ag, these electrons acquire enough potential to react with oxygen in water to produce a significant amount of reactive oxygen species, like superoxide radicals (as shown by the confirmatory tests for reactive oxygen species), leading to the oxidation of the pollutants followed by their mineralization. The photogenerated holes further help in the direct oxidation of the pollutants or by indirect oxidation via the formation of ROS, which decompose the pollutants into minerals, as evidenced by scavenging tests. Thus, Ag-CZFO/ZnO can efficiently utilize solar energy for the degradation of ALR.

**Figure 14:**
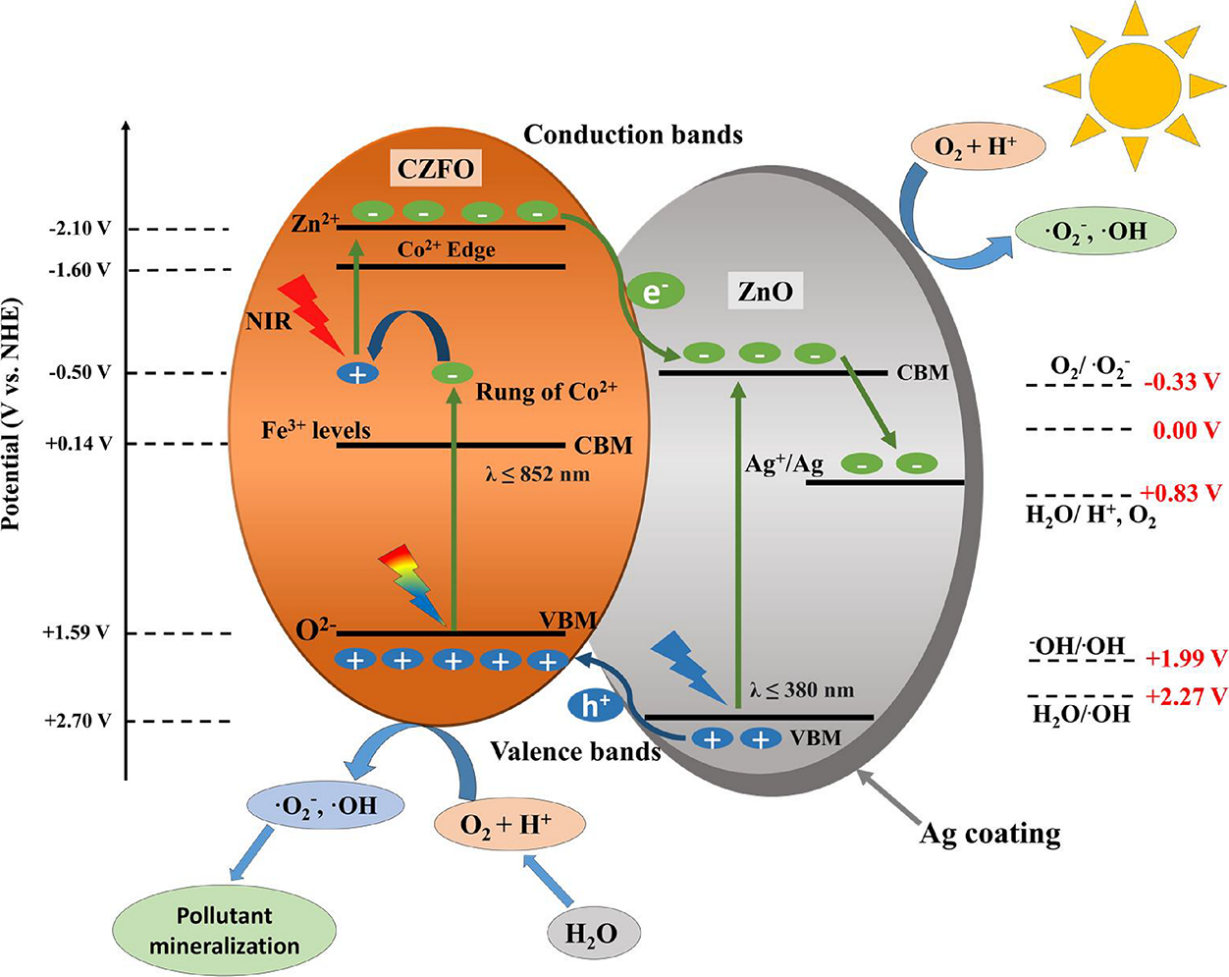
Schematic of the mechanism of pollutant degradation over Ag-CZFO/ZnO under (visible + NIR light).

A comparative analysis of our work with previously published studies on Allura Red AC (ALR) dye degradation is presented in Table 1.^16^ The key findings, such as variations in photocatalyst dosage, degradation duration, initial dye concentration, catalyst reusability, and overall degradation efficiency, are displayed. Our study distinguishes itself by employing a notably higher initial concentration of ALR dye (24.8 mg/L) for degradation experiments than previously reported, demonstrating robust catalytic performance under more challenging conditions. Moreover, all photodegradation experiments were conducted at the natural pH of the dye solution (pH = 5.89) without pH adjustment, achieving an impressive degradation efficiency of 96%. This contrasts with other studies, which typically required pH modification to enhance the degradation rates. The adsorption–desorption equilibrium was carefully established, providing rigorous conditions for the photocatalytic assessment.

**Table 1:** Comparison of ALR Photodegradation Using Different Reported Photocatalysts

Sr. no.	Photocatalyst	ALR initial conc. (*C*_0_) (mg L^–1^)	pH	Amount of catalyst (g L^–1^)/time (min)	Time for adsorption desorption equilibrium (min)	Degradation (%)	Region of solar spectrum used	Intensity of light at the sample	Reusability study	ref.
1	UiO-66-NDC	20.0	5–6	0.3/45	30	96	UV light	4.2 mW/cm^2^	88% (5th cycle)	^50^
2	g-CN with amino acid functionalization	10.0	7	0.5/35	30	93.8	sunlight		76.3% (6th cycle)	^51^
3	Fe_3_O_4_/HAP/Ag (With oxidant addition)	20.0	10	–/40	30	98.4	visible light			^52^
4	CaWO_4_/ g-C_3_N_4_	30.0	6	0.6/120	30	79.4	visible light	150 W (at source)	73.6% (5th cycle)	^37^
5	MWCNT-TiO_2_–Ag	5.0	6	0.5/160	60	79.9	UV light	15 W (at source)	78.0% (3rd cycle)	^53^
6	Polyaniline/TiO_2_	19.8	2	–/80		72.0	UV light	15 W (at source)		^54^
7	NiO	10.0		0.5/90	30	91.7	UV light		89.2% (3rd cycle)	^55^
8	F–Fe–TiO_2_/SiO_2_	10.0	3	flow reactor/720		89.5	visible light	400 W (at source)	63.0% (7th cycle)	^56^
9	TiO_2_ (Immobilized)	5.0		flow reactor/300		58.0	UV light	2.72 mW (at source)		^57^
10	nickel–cobalt oxide nanosheets	5.0		0.5/10	120	94.7	UV light		90.0% (10th cycle)	^58^
11	ZnO-PbS	5.0	5	0.5/45	5	93.0	Xe lamp	35 W (at source)	82.2% (4th cycle)	^59^
12	Ag-CZFO/ZnO	24.8	5.89	1/150	60	96	visible + NIR	visible light: 167 W/m^2^ NIR: 4319 W/m^2^	92.2% (5th cycle)	this work

A detailed reusability study showed excellent stability, with 92.2% degradation maintained, even after five successive cycles. The current work introduces a novel laddered type-I heterojunction of earth-abundant and nontoxic photocatalysts, featuring selective Ag coating on CZFO/ZnO interfaces without the need for any reducing agent. This configuration efficiently exploits the localized surface plasmon resonance (LSPR) effect and enables broad-spectrum light absorption (UV, visible, and NIR), thus maximizing solar energy utilization. Importantly, while most existing literature relies exclusively on UV irradiation, our photocatalyst system harnesses the full solar spectrum, facilitating ALR dye degradation under practical sunlight conditions.

Collectively, these advancements of high initial dye loading, natural pH operation, strong reusability, and broad-spectrum solar activation demonstrate a substantial advancement in performance and sustainability over previous approaches. The use of cost-effective, environmentally safe materials lowers the overall treatment cost and highlights the promise of this methodology for scalable water remediation and industrial applications.

## 4 Conclusions

This study systematically investigated the photocatalytic degradation of Allura Red AC (ALR), a model azo dye pollutant, under visible and near-infrared light irradiation. 96% dye could be degraded under optimized conditions (50 μM ALR, catalyst dosage: 1 g/L, and pH: 5.89), within 150 min using visible light. The observed rate constant for this degradation was 0.0188 min^–1^. The degradation process was notably accelerated when an electron scavenger, hydrogen peroxide (H_2_O_2_), was added, increasing the rate constant to 0.0476 min^–1^. In contrast, the inclusion of a hole scavenger, methanol (CH_3_OH), inhibited the reaction, highlighting the essential role of photogenerated holes in the oxidation pathway. The ROS, such as hydroxyl and superoxide radicals, were identified as the main oxidizing agents using terephthalic acid and nitro blue tetrazolium probe techniques. The effective reduction of COD from 31.0 to less than 5 ppm confirmed the complete mineralization of the dye rather than just a partial transformation. The nanocomposite displayed 100-fold enhancement in the photocurrent, attesting to significantly decreased electron–hole recombination. The Nyquist plots displayed the smallest semicircle, confirming the lowest interfacial resistance and the fastest charge transfer process. The plasmonic laddered heterojunction mechanism of our photocatalyst undoubtedly establishes the superiority of our solar photocatalyst. Additionally, the photocatalytic system demonstrated excellent reusability, maintaining a degradation efficiency of over 92% after five successive cycles. Overall, these findings underscore the effectiveness and reliability of the developed photocatalytic method for degrading persistent azo dyes. With its broad-spectrum light responsiveness, reusability, and capability for complete mineralization, this system shows promising potential for practical applications in wastewater treatment targeting resistant organic pollutants.
